# Evaluation of a mHealth Data Quality Intervention to Improve Documentation of Pregnancy Outcomes by Health Surveillance Assistants in Malawi: A Cluster Randomized Trial

**DOI:** 10.1371/journal.pone.0145238

**Published:** 2016-01-05

**Authors:** Olga Joos, Romesh Silva, Agbessi Amouzou, Lawrence H. Moulton, Jamie Perin, Jennifer Bryce, Luke C. Mullany

**Affiliations:** 1 Institute for International Programs, Department of International Health, Johns Hopkins Bloomberg School of Public Health, Baltimore, Maryland, United States of America; 2 Statistics Division, Economic and Social Commission for Western Asia, United Nations, Beirut, Lebanon; 3 UNICEF, New York, New York, United States of America; 4 International Center for Maternal and Neonatal Health, Department of International Health, Johns Hopkins Bloomberg School of Public Health, Baltimore, Maryland, United States of America; University of Tennessee Health Science Center, UNITED STATES

## Abstract

**Background:**

While community health workers are being recognized as an integral work force with growing responsibilities, increased demands can potentially affect motivation and performance. The ubiquity of mobile phones, even in hard-to-reach communities, has facilitated the pursuit of novel approaches to support community health workers beyond traditional modes of supervision, job aids, in-service training, and material compensation. We tested whether supportive short message services (SMS) could improve reporting of pregnancies and pregnancy outcomes among community health workers (Health Surveillance Assistants, or HSAs) in Malawi.

**Methods and Findings:**

We designed a set of one-way SMS that were sent to HSAs on a regular basis during a 12-month period. We tested the effectiveness of the cluster-randomized intervention in improving the complete documentation of a pregnancy. We defined complete documentation as a pregnancy for which a specific outcome was recorded. HSAs in the treatment group received motivational and data quality SMS. HSAs in the control group received only motivational SMS. During baseline and intervention periods, we matched reported pregnancies to reported outcomes to determine if reporting of matched pregnancies differed between groups and by period. The trial is registered as ISCTRN24785657.

**Conclusions:**

Study results show that the mHealth intervention improved the documentation of matched pregnancies in both the treatment (OR 1.31, 95% CI: 1.10–1.55, p<0.01) and control (OR 1.46, 95% CI: 1.11–1.91, p = 0.01) groups relative to the baseline period, despite differences in SMS content between groups. The results should be interpreted with caution given that the study was underpowered. We did not find a statistically significant difference in matched pregnancy documentation between groups during the intervention period (OR 0.94, 95% CI: 0.63–1.38, p = 0.74). mHealth applications have the potential to improve the tracking and data quality of pregnancies and pregnancy outcomes, particularly in low-resource settings.

## Introduction

The field of global health has set ambitious goals, including the Millennium Development Goals and the recently defined Sustainable Development Goals [1]. These initiatives have catalyzed donor support and country engagement to make progress on education, health, and socio-economic development [2]. Shortages of skilled health workers and demanding scale-up agendas to meet targets have focused attention on the potential of community health workers (CHWs) to extend the reach of the formal health system [3]. Although CHWs are a heterogeneous group of health professionals globally, a common element is their presence within the community they serve. CHWs are gaining job responsibilities, recognition, and financial compensation due to skilled health worker shortages [4–7]. Their community presence has the potential to minimize access and utilization barriers by bringing health care services closer to the population they serve [3,8]. Reliance on CHWs for multiple responsibilities, however without commensurate consideration of appropriate support or compensation, can be demotivating and affect their performance [9, 10].

Researchers have demonstrated the importance of supervision and incentives to improve motivation and performance among CHWs [11–13]. Mobile technology for health, commonly called mHealth, is a growing field in public health that uses mobile technology for health care delivery, improving efficiency and data quality, minimizing bottlenecks, and supporting continuous education and adherence [14–17]. mHealth applications are diverse and creative, although most fall within one of twelve domains: client education; point of care diagnostics; vital event tracking; data collection; electronic health records; decision support; provider communication; provider work planning; provider training; human resource management; supply chain management; and financial transactions [18]. mHealth applications are relatively new, so many mHealth projects to date have been implemented as pilots, and rigorous evaluations are only recently emerging [19–21]. Conclusions on effectiveness vary and research gaps remain, especially for rigorous quantitative evaluations, cost-effectiveness studies, and assessments focused on scaling up projects beyond the pilot stage [22,23].

The Institute for International Programs (IIP) at Johns Hopkins University and the Malawi National Statistical Office (NSO) collaborated to implement a community-based vital event documentation system (“Real-time Mortality Monitoring”, RMM) using Health Surveillance Assistants (HSAs), government trained and paid CHWs with a scope of work set by the Malawi Ministry of Health [24]. We implemented the RMM project in two districts in Malawi from January 2010 through December 2013 to assess the completeness and accuracy of under-five mortality reporting by HSAs. Details of this study are described elsewhere [25–28].

An estimated 45% of under-five deaths occur in the neonatal period and many of these deaths are not reported due to cultural and social norms [29–31]. To promote HSA surveillance of pregnant women and families with children, we designed RMM to include pregnancy tracking to facilitate the capture of neonatal deaths. To maintain motivation of HSAs and encourage high data quality, we provided incentives and supports. One such support was a mHealth intervention with two levels of intensity; these levels were randomly allocated within clusters to HSAs. We present results from this cluster randomized mHealth intervention designed as a job aid to improve the documentation of matched pregnancies. We defined matched pregnancy documentation as a recorded pregnancy with a matched outcome: abortion, miscarriage, stillbirth, live birth, or the out-migration of the pregnant mother.

## Methods

### Study population

We conducted the data quality mHealth intervention with the RMM project implemented in the Balaka and Salima districts of Malawi. The intervention period for the mHeath trial was from November 2012 to November 2013, and the intervention period for the RMM project was from January 2010 to December 2013. The districts in the RMM project were selected for their high under-five mortality, high fertility, ease of access for the study team, average population size relative to other districts in the country, and full coverage by HSAs deployed in the district. Each HSA in Malawi is assigned to a catchment area of approximately 1,000 inhabitants and its associated health facility, covering a radius of eight kilometers except in district-defined hard-to-reach catchment areas [32]. We randomly selected 160 catchment areas: 80 from among 280 catchment areas in Balaka and 80 from among 355 catchment areas in Salima, Details of the randomization are presented elsewhere [25]. Among the selected catchment areas, the average number of HSAs affiliated with a health facility was 5.2 (range: 1 to 19). The selected HSAs were associated with a total of 30 health facilities. All HSAs assigned to RMM catchment areas were eligible for inclusion in the mHealth intervention.

Through the RMM project, HSAs received training on the documentation of pregnancies, births, and deaths, a task within the HSA scope of work set by the Ministry of Health [33]. At the start of the RMM project, HSAs and supervisors received a mobile phone to facilitate communication with RMM project staff for data editing calls and field visit notifications. Due to poor results in the midline assessment of under-five mortality reporting, we implemented phase two of the RMM project in September 2012 with additional incentives and data quality supports to improve HSA documentation of vital events.

### Design

**Fig 1:**
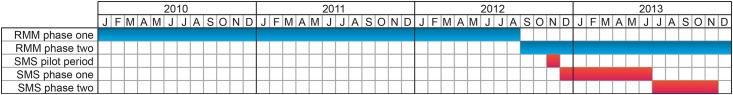
SMS timeline. Timeline of RMM project and SMS implementation from January 2010 through December 2013.

We designed the intervention as a mobile support for HSAs using one-way short message services (SMS) sent by the mHealth coordinator at the NSO to HSAs in the RMM project. The mHealth intervention had a three-week pilot phase and a twelve-month implementation phase divided into two phases. The study team modified the intervention after eight months of implementation to incorporate feedback from the HSAs suggesting that the variety of SMS and their frequency should be increased (Fig 1). The intervention is described in two phases. Phase one ran for seven months between December 2012 and June 2013. Phase two ran for five months from July to November 2013. Throughout both phases, the treatment group received high-intensity SMS with motivational and data quality content based on the RMM data quality guidelines (Table 1). The control group received minimal-intensity SMS with basic motivational content. S1 File presents all SMS sent to the control group and a selection of the SMS sent to the treatment group during each phase of the intervention [34].

**Table 1:** SMS intervention two-phase schedule.

	Phase one	Phase two
**Time period**	12/2012–6/2013	7/2013–11/2013
*Weeks*	34	26
**Total SMS sent**		
*Control group*	2 SMS/week	2 SMS/week
*Treatment group*	3 SMS/week	5 SMS/week
**SMS content**		
*Control group*	Motivation	Motivation
*Treatment group*	Data quality and motivation	Data quality and motivation
**SMS message variety**		
*Control group*	3 unique messages	6 unique messages
*Treatment group*	12 unique messages	24 unique messages
**Average weekly airtime US$**	$19.00	$30.22
**Total airtime US$**	$646.11	$785.70

For both periods, we pretested the SMS among purposively selected HSAs (only in Balaka for phase one SMS, in both districts for phase two SMS). To meet the 160-character limit of the SMS, established abbreviations were used and punctuation dropped in ways that pilot testing confirmed did not affect the HSAs’ comprehension of the message. For both treatment and control groups, the SMS were randomized and scheduled as per the schedule. The repetition of SMS presented in a randomized sequence was done to reinforce important data quality messages in the treatment group yet avoid alert fatigue.

We selected Frontline SMS as the messaging platform because it is open source and fit the technical and management capacities of the IIP and NSO data management team [35]. Frontline SMS functions with a GSM wireless modem and SIM card for which we selected Airtel, one of the two leading telecom operators in Malawi at the time of the study design. Because HSAs had Airtel SIM cards in their RMM-issued mobile phones, this allowed us to pay the lowest airtime costs.

Prior to the start of the intervention, the lead mHealth researcher trained two RMM data management team members at the NSO to serve as field coordinators for the study. During phase one, the two field coordinators alternated responsibility each week. During phase two, one coordinator retained lead responsibility and was supported by the other coordinator when conducting field visits. The lead mHealth researcher supervised the initial implementation of the intervention, maintained daily communication with the mHealth coordinators throughout the intervention, and assisted in resolving technical and logistical challenges.

At the September 2012 data review meetings of the RMM project, the NSO data management team introduced the mHealth intervention as a mobile support to HSAs and supervisors. We did not train HSAs in sending or reading SMS, because SMS were an established method of communication in the RMM project. At the start of the SMS pilot period and the second phase of the mHealth intervention, the field coordinator sent SMS with a brief description of the intervention expectations and schedule.

Prior to each scheduled transmission of SMS to HSAs, the field coordinator sent a test SMS to three NSO RMM team members to ensure proper functioning of the Frontline SMS platform. Once receipt of the test SMS was confirmed by at least one of the recipients of the test SMS, the field coordinator sent the scheduled SMS. For HSAs with two phones, the field coordinator sent the same scheduled SMS to both numbers to increase the likelihood that the SMS was received. After the field coordinator sent the SMS, the Frontline SMS platform was kept open and the USB GSM modem was kept connected in the event that an HSA responded with a question or clarification.

Although the intervention was unidirectional, HSAs occasionally sent response SMS to the field coordinators. The field coordinator documented SMS communications and airtime costs each day SMS were sent and tallied totals at the end of the week (Table 1). Each week, the lead mHealth researcher and field coordinators communicated and confirmed the SMS schedule, the number of SMS that had been sent, responses received from HSAs, and weekly airtime costs, all of which were recorded in the SMS program management database in Microsoft Excel.

As per the scope of work set by the Malawi Ministry of Health, HSAs track pregnancies, births, and deaths using their Village Health Registers (VHRs). For the RMM project, we asked HSAs to extract these events monthly onto a RMM extraction form that was submitted to the NSO. Details of the data collection process are available elsewhere [25,27]. The data editor reviewed these forms and called HSAs who had reported an adverse event (abortion, miscarriage, stillbirth, maternal death, or neonatal death) to confirm the event and verify the outcome classification.

The primary outcome measure was the improvement in matched pregnancy documentation between groups during the intervention period. Possible pregnancy outcomes included adverse events (abortion, miscarriage, stillbirth), live birth, and out-migration of the pregnant mother. The secondary outcome measures were the improvements in matched pregnancy documentation by group between baseline and intervention periods. We also evaluated changes in matched pregnancy documentation by HSA residency in their assigned catchment area.

Pregnancies and outcomes were matched using the six-digit HSA code and the woman’s unique 11-digit ID. Matching results included three outcomes: (i) pregnancies matched to an outcome (ii) live births and adverse pregnancy outcomes without a pregnancy match (iii) pregnancies without a matched outcome. Only matched (i) and unmatched pregnancies (iii) were used to analyze the change in documentation of matched pregnancies, our primary and secondary outcome measures. We exclude unmatched outcomes (ii) from the analysis since they can be used to address a different research objective, the characteristics of women missed by HSAs during pregnancy.

### Statistical analysis

**Fig 2:**
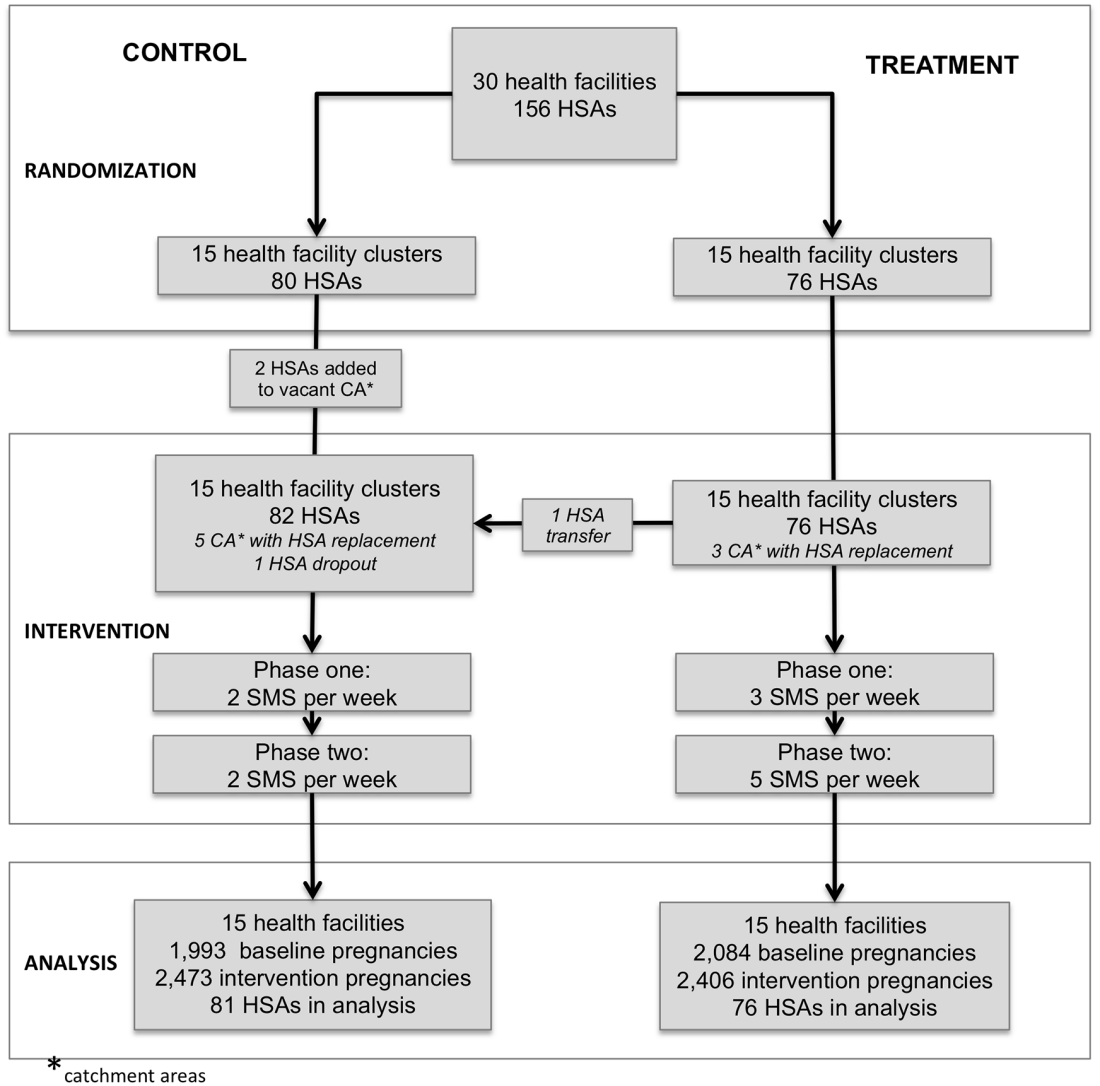
Trial profile. Profile of trial clusters at randomization, intervention, and analysis periods.

We were restricted to the sample size of the RMM project, i.e. the number of pregnancies and outcomes that occurred within 160 randomly selected catchment areas of Balaka (n = 80) and Salima (n = 80) districts affiliated with thirty health facilities and 160 HSAs. We included only 156 of the 160 catchment areas in the randomization since four catchment areas did not have HSAs at the time of randomization (Fig 2). We randomized at the level of the cluster, health facilities (n = 30). We did not use individual-level randomization to prevent contamination from HSA collaboration and interaction at their associated health facility.

We calculated the study power using published data from comparable studies conducted in Africa. We estimated that HSAs documented pregnancy outcomes for 70% of pregnancies at baseline, and HSAs in the control group would maintain this percentage during the intervention period [36]. We estimated HSAs in the treatment group would improve by 20% to document outcomes in 90% of the reported pregnancies [21]. Assuming a baseline prevalence of 70%, an effect size of 20%, a confidence level of 95%, an estimated intracluster correlation (ICC) of 0.3, inclusion of 30 clusters (15 control, 15 treatment), and a two-sided alpha of 0.05, we estimated a study power of 0.54, which falls short of the recommended power of 0.80 to reject the null hypothesis of no impact. This power estimate did not include pregnancy matching prevalence or ICC from baseline data, or factor variability in cluster size. Although we recalculated power using baseline data to reach a favorable estimate, we assume the study power of 0.54 given the study was designed with this estimate [37–39] (S2 File).

To improve balance in treatment and control arms, we stratified by district and used a +/- 10% relative restriction to constrain on three variables: average catchment area population per HSA; total cluster population; and baseline timeliness of extraction form submission. The validity of the acceptable randomizations was checked by evaluating the number of times any two randomization units appeared in the same arm together [40]. From the list of 5,000 acceptable randomization schemes, one was randomly selected and a Bernoulli trial was used to determine the arm allocated to the intervention [41].

The data clerks performed double data entry in CSPro on edited forms [42]. Abortions, miscarriages, and stillbirths were recorded in an adverse outcomes database maintained by the data editor (doi: 10.7281/T1F769G3). HSA characteristics included in the mHealth intervention analysis were collected in August 2013 through the Village Health Register Verification assessment conducted for the RMM project. In catchment areas with HSA turnover, characteristics of the HSA interviewed for the assessment were used.

We did not conduct intention-to-treat analysis among HSAs who left RMM or transferred between RMM catchment areas, in order to minimize contamination in groups. HSAs who moved from a health facility cluster in one group to a health facility cluster in other treatment group received messages consistent with their new group. HSAs who transferred out of their RMM catchment area were instructed to stop submitting RMM extraction forms and were dropped from the mHealth intervention. Their truncated data was included in the analysis. HSAs were masked to their treatment group allocation. It was not possible to mask the field coordinators to treatment group allocation since they were responsible for maintaining an updated contact list on Frontline SMS.

We used a population-averaged panel data model with generalized estimating equations to analyze the effect of the mHealth intervention on matched pregnancy documentation using Stata 12 [39]. The model assumed an independence working structure among observations. We specified a binomial distribution for the outcome variable and a logistic link between the outcome variable and predictor. We included clusters defined by health facility catchment area to account for the clustered design. Baseline and intervention data were included, so we specified an interaction effect between the intervention period and treatment group. We only considered explanatory variables that were not included in the constrained randomization. We evaluated district location and catchment area residence as explanatory variables, and only included HSA catchment area residence in the final model. We dropped unmatched pregnancies that would not have reached eight months’ gestation in October 2013 or earlier. These unmatched pregnancies did not have full matching eligibility with the RMM dataset, because the dataset only included events through December 2013.

### Ethical review

We obtained ethical approval in the U.S. from the Institutional Review Board (IRB) at the Johns Hopkins University Bloomberg School of Public Health, and in Malawi from the National Health Sciences Research Committee. We obtained a waiver of written consent from the IRB. Approval letters are available upon request.

## Results

In the initial design, 76 HSAs affiliated with 15 health facilities were allocated to the treatment group and 80 HSAs affiliated with 15 health facilities were allocated to the control group (Fig 2). Two HSAs filled vacant catchment areas in the control group after randomization and were included in the study. Among the 158 HSAs linked to 30 health facilities in the RMM study, all HSAs participated in the mHealth intervention.

During the intervention, one HSA in the control group left the RMM project. Eight catchment areas experienced HSA turnover. One HSA replacement included the transfer of an HSA from a catchment area in the treatment group to a catchment area in the control group. The analysis was conducted with and without catchment areas with HSA changes. There were no substantive differences between the two sets of results, and the final analysis includes catchment areas with HSA changes during the intervention period.

**Fig 3:**
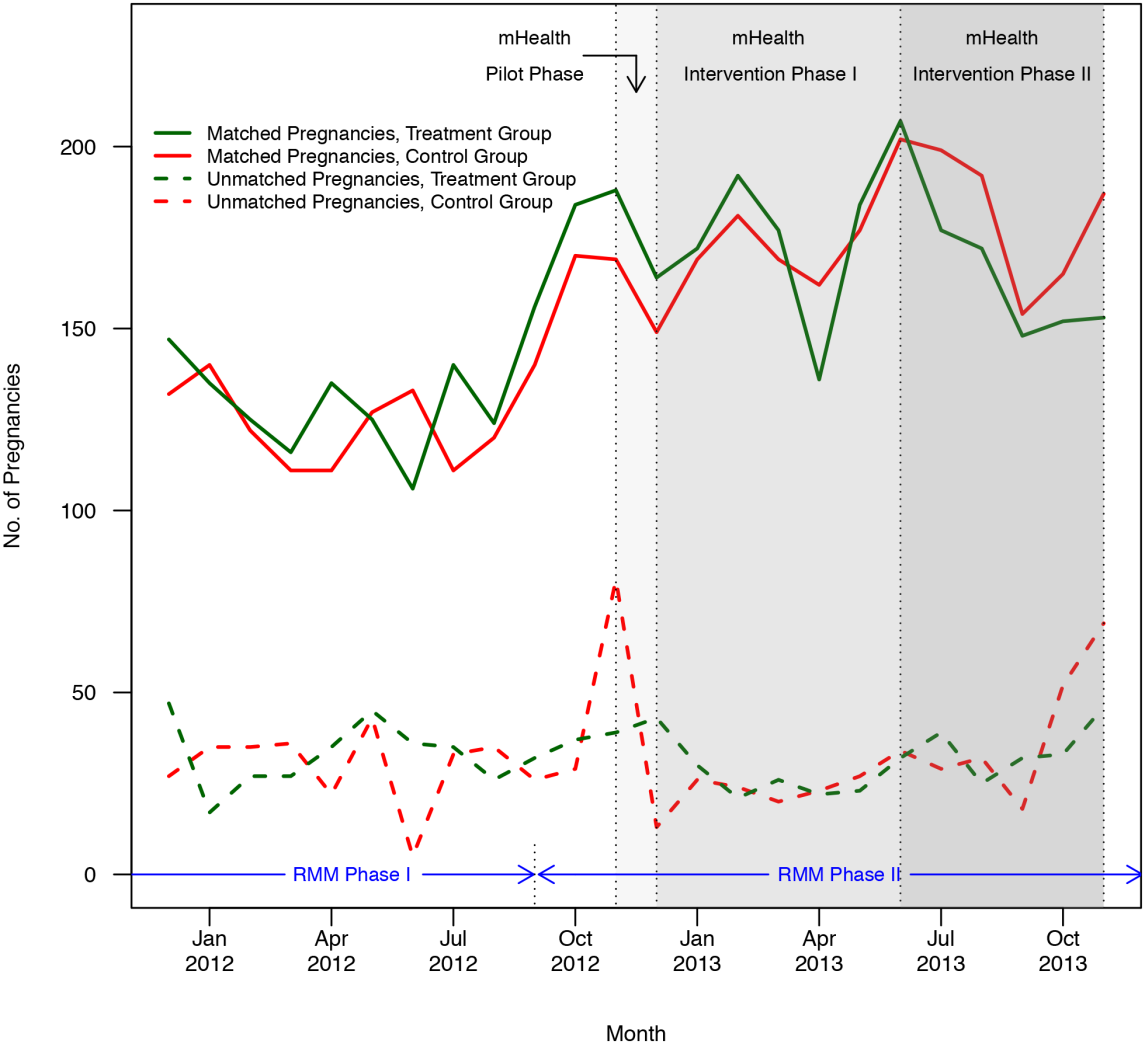
Matched and unmatched pregnancies by group. Number of matched and unmatched pregnancies by group during SMS baseline and intervention periods from December 2011 through November 2013.

**Fig 4:**
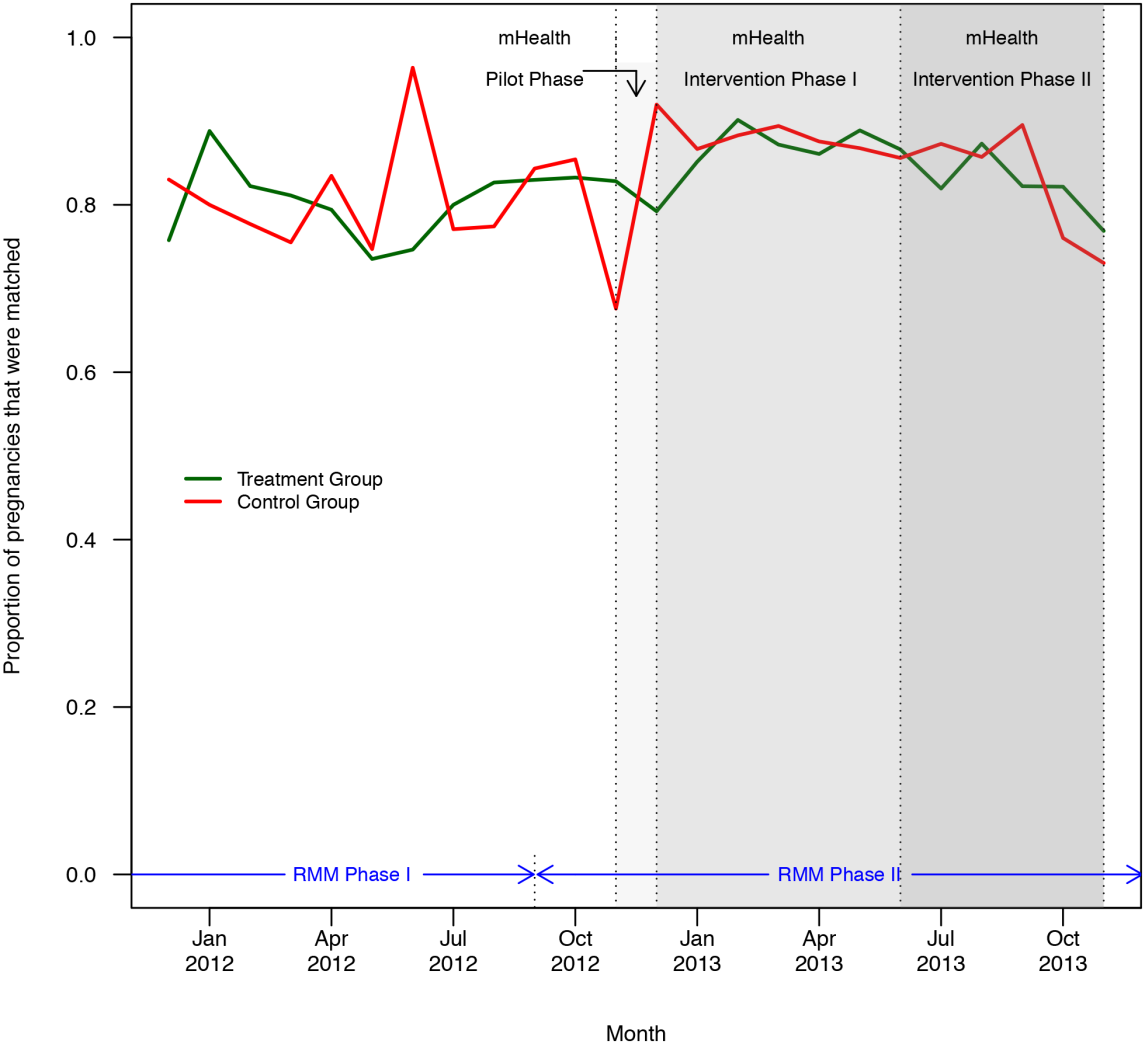
Proportion of matched pregnancies by group. Proportion of matched pregnancies by group during SMS baseline and intervention periods from December 2011 through November 2013.

Table 2 presents treatment and control group characteristics. HSA sex and catchment area population were used to constrain the randomization. Baseline characteristics of HSAs are similar across groups, with only a small difference in the percentage of HSAs with large or small catchment areas. HSA reporting of matched pregnancies increased at the start of RMM phase two in September 2012, and was generally maintained over time. However, there was a jump in unmatched pregnancies at the end of the mHealth intervention (Fig 3). The monthly proportion of pregnancies that were matched increased in both groups during most of the mHealth intervention period (Fig 4). The proportion in both groups appears to decrease in September 2013, which coincides with the time-intensive preparations for the large endline survey conducted by the RMM data management team. During this period, the RMM data management team had limited day-to-day engagement with HSAs and RMM field members because they were preoccupied with preparations for the endline survey.

**Table 2:** HSA characteristics by group.

	Control		Treatment		Total	
	n	%	n	%	n	%
**Health facilities** *(n = 30)*						
*Balaka*	6	40%	7	47%	13	43%
*Salima*	9	60%	8	53%	17	57%
**HSAs working on RMM** *(n = 158)*						
*Female*	25	30%	26	34%	51	32%
*Male*	57	70%	50	66%	107	68%
**Catchment area posting**: *(n = 157)*						
HSA does not reside in CA*	43	53%	41	54%	84	54%
HSA does reside in CA*	38	47%	35	46%	73	46%
**Catchment area size**: *(n = 158)*						
*<900 inhabitants*	11	13%	4	5%	15	9%
*900–1100 inhabitants*	19	23%	20	26%	39	25%
*1101–1400 inhabitants*	26	32%	33	43%	59	37%
*≥1400 inhabitants*	26	32%	19	25%	45	28%

*catchment area

During the baseline and intervention periods, HSAs reported 7,407 matched pregnancies, of which 4,140 were reported in the intervention period (55.9%). Table 3 summarizes the number of events reported by group and the distribution of event types. Treatment group HSAs reported slightly more matched pregnancies at baseline than the control group (1,681 vs. 1,586). Both groups reported a similar percentage of live births among matched pregnancies in both periods. In the baseline period, the control group reported no abortions. The five abortions reported by the treatment group only accounted for 0.3% of its reported events in the baseline period. Control group HSAs reported more out-migration than HSAs in the treatment group, with the difference increasing slightly in the intervention period (1.8% vs. 1.4% and 3% vs. 2.1%, respectively). Treatment group HSAs reported twice as many adverse pregnancy outcomes in the intervention period as the control group HSAs. The absolute number of events increased during the intervention period, but the overall distribution of events remained unchanged.

**Table 3:** Matching results by intervention periods and groups.

	Control		Treatment		Total	
	n	%	n	%	n	%
**All documented events**						
*Unmatched pregnancies*	774	15.1%	775	14.3%	1,549	14.7%
*Unmatched outcomes*	673	13.1%	946	17.4%	1,619	15.3%
*Matched pregnancies*	3,692	71.8%	3,715	68.3%	7,407	70.0%
**Total**	5,322	100.0%	5,612	100.0%	10,575	100.0%
**Outcomes of matched pregnancies**						
**Baseline period**						
*Out-migration of pregnant woman*	28	1.8%	24	1.4%	52	1.6%
*Abortion*	0	0.0%	5	0.3%	5	0.2%
*Miscarriage*	8	0.5%	7	0.4%	15	0.5%
*Stillbirth*	20	1.3%	28	1.7%	48	1.5%
*Live birth*	1,530	96.5%	1,617	96.2%	3,147	96.3%
**Total**	1,586	100.0%	1,681	100.0%	3,267	100.0%
**Intervention period**						
*Out-migration of pregnant woman*	64	3.0%	43	2.1%	107	2.6%
*Abortion*	3	0.1%	7	0.3%	10	0.2%
*Miscarriage*	12	0.6%	21	1.0%	33	0.8%
*Stillbirth*	36	1.7%	58	2.9%	94	2.3%
*Live birth*	1,991	94.5%	1,905	93.7%	3,896	94.1%
**Total**	2,106	100.0%	2,034	100.0%	4,140	100.0%

Table 4 summarizes the analysis of the primary and secondary outcomes. The intervention did not result in statistically significant improvements in matched pregnancies between groups during the intervention period (OR 0.94, 95% CI: 0.63–1.38, p = 0.74). However, we found statistically significant differences by groups between baseline and intervention periods. HSAs in the control group reported 46% more matched pregnancies during the intervention period as compared to the baseline period (95% CI: 1.11–1.91, p = 0.01). HSAs in the treatment group reported 31% more matched pregnancies during the intervention period as compared to the baseline period (95% CI: 1.10–1.55, p<0.01). We noted statistically significant differences in matched pregnancy documentation by catchment area residency. HSAs who do not reside in their catchment area are 28% more likely to match pregnancies than HSAs who reside in the catchment area (95% CI: 0.54–0.96, p = 0.03).

**Table 4:** Odds ratio by intervention periods and groups.

	Odds Ratio	95% CI	p value
**Primary outcome**			
Control group-intervention period	*reference*		
Treatment group- intervention period	0.94	0.63 to 1.38	0.74
**Secondary outcomes**			
Control group- baseline period	*reference*		
Control group- intervention period	1.46	1.11 to 1.91	0.01
Treatment group- baseline period	*reference*		
Treatment group- intervention period	1.31	1.10 to 1.55	<0.01
**Catchment area residency**			
Does not reside in catchment area	*reference*		
Resides in catchment area	0.72	0.54 to 0.96	0.03

## Discussion

This mHealth intervention, designed and analyzed at the cluster level, led to an improvement in HSA documentation of matched pregnancies in each group between baseline and intervention periods. However, we did not find a statistically significant difference in matched pregnancy documentation between groups during the intervention period. Overall documentation of pregnancies and outcomes improved during the intervention period, with notable increases in the documentation of out-migration and adverse pregnancy outcomes.

A range of factors can influence CHW performance, but translating these factors to mobile applications is novel and results vary [20,43]. Researchers evaluating timeliness of reporting by CHWs have found that it can be improved with reminders by phone or SMS [17,44]. DeRenzi et al. found that supervisor follow-up via an SMS reminder system was associated with improved timeliness. SMS reminders alone did not improve timeliness. Our study was limited to SMS sent only to HSAs, without the inclusion of supervisors, who had higher rates of turnover in RMM than HSAs. The results of Derenzi et al. are notable in light of research on the importance of supervision on CHW motivation, which is a strong influence on performance [9,13,17]. Furthermore, they demonstrate that the design of a mHealth intervention can influence effectiveness. We pre-tested the SMS and made deliberate changes to the initial design of the SMS intervention based on HSA feedback for more variety of SMS content and increased frequency. Robust formative research elaborating recipient perceptions of SMS intervention design and feedback mechanisms may facilitate the design and implementation of an effective mHealth intervention specific to the needs of the SMS recipients [45,46].

The improvements in the control group were not expected based on research showing no effect with simple, repetitive SMS [47]. We tailored the high-intensity intervention SMS to match the data quality guidelines of the RMM project since research has shown improved efficacy of tailored and personalized SMS [48]. Qualitative studies have found that mHealth interventions have had an unintended motivational and empowering effect on CHWs [45,49]. Additionally de Tolley et al. found statistically significant improvements with motivational SMS, though not with informational SMS sent to improve uptake of HIV counseling and testing [50]. These results may explain the statistically significant improvement in the documentation of matched pregnancies among control group HSAs in the intervention period compared to the baseline period. Qualitative assessments with multiple evaluation points to capture motivation levels and perceptions of mHealth content and frequency should be conducted to explain such unexpected results. Future mHealth research should be multidimensional, using qualitative research to complement and explain quantitative results [20].

The increase in the documentation of abortions, miscarriages, and stillbirths is noteworthy, because these events are difficult to capture in low-income countries [30,51]. Haws et al. found that women in Tanzania did not share pregnancy loss or neonatal death with their community [31]. Research in South Africa and Ghana found that women hide pregnancies to protect against witchcraft [52,53]. Given this level of discretion, CHWs may be in a strong position to document these hard-to-capture events, given their presence in the community and their role as service providers. The increase in documented adverse pregnancy outcomes during the intervention period by both groups, and notably the treatment group receiving data quality SMS about these sensitive events, warrants further attention.

In a study conducted among HSAs in the Mwanza district of Malawi, Kok & Muula found that HSAs identified transportation challenges as one of the main work dissatisfiers [54]. Transportation challenges have also been identified as a contextual factor that can impact CHW performance [55]. Our results counter performance results from other studies. We found that HSAs who do not reside in their catchment area are more likely to document matched pregnancies than HSAs who reside in their catchment area. Catchment area residence is only one metric to assess HSA presence. It does not consider distance of the residence from catchment area or HSA time spent in the catchment area, variables that might be more indicative of community presence. Further research should be conducted to evaluate CHW community presence and the best metrics for assessment.

A major limitation to the study is limited power of the study due to a restricted study population. However, this design allowed for a pre-post intervention design with complete baseline data. This also allowed calculation of three ICC coefficients that can be used by researchers designing clustered studies. Another limitation is the change in design of the intervention midway through the trial to increase the dose of SMS in the treatment group from three SMS a week to five. We made this decision with great consideration. We agreed that the modest increase in SMS frequency in the intervention group responded to the needs of the HSAs. The change in intervention may have introduced bias for assessing the intervention effect. We expect the bias to be positive relative to phase I results and negative for phase II results in the treatment group assuming the increased dose improved matched pregnancy documentation. We did not measure the effect of the change due to the underpowered study design and absence of notable differences between phases in both treatment groups. This further highlights the importance of qualitative assessments in complementing quantitative results to elucidate HSA perceptions on the effect of any form of SMS modifications [20,45].

The Frontline SMS system did provide confirmation that SMS were sent though the cellular network, but we did not have confirmation from HSAs that the message was received, read, and understood, or about the delay between the arrival of the SMS and its reading. This required the assumption that a SMS confirmed by Frontline was received and read by the HSA in a timely manner. A bidirectional SMS intervention would have allowed us to confirm receipt and quantify delays, but the intervention would no longer be a reminder system and require a higher level of engagement from the field coordinator at the NSO and from the HSAs. If this intervention is reconsidered for HSAs in Malawi, researchers should consider pilot testing a bidirectional system to determine its feasibility and acceptability given the more interactive design.

This intervention was not designed to be scaled up as implemented through the RMM project. New investments would be required to maintain the intervention and to scale it up. This pilot study is an example of the “*pilotitis”* that is plaguing mHealth interventions, but also generated important information that can be used to develop an improved design prior to scale-up [56].

The ubiquity of mobile phones and growing access to mobile networks in hard-to-reach areas makes mHealth a very attractive tool, especially in low-income countries. Our results reveal that regularly scheduled SMS with basic motivational messages, both with and without data quality content, can improve performance of CHWs in the documentation of pregnancy outcomes. The study was underpowered so results should be interpreted with caution. These results are still important given the growing interest in mHealth applications and respond to calls for their rigorous evaluation [57]. Researchers, program planners, and policy makers stand to benefit from sharing results from mHealth applications.

## Conclusions

We observed measurable improvements in the documentation of matched pregnancy outcomes associated with regular, unidirectional SMS of two intensity levels sent to CHWs in rural Malawi. Improvements were noted in each group regardless of whether or not the motivational SMS additionally included content explicitly related to data quality. The study was underpowered so results should be interpreted with caution. We did not find a difference in the documentation of matched pregnancies between groups during the intervention period. The effect of continuous guidance and support to CHWs via SMS on the data quality of pregnancy outcomes should be further investigated using randomized field studies. In particular, further research is needed to exploit the motivational effect of SMS communications, their content, and frequency. We believe that mHealth tools have the potential to improve the tracking of pregnancies and the documentation of pregnancy outcomes, particularly in low-resource settings.

## Supporting Information

S1 File Selection of SMS sent to RMM HSAs during phases one and two. (DOCX)

S2 File Power and ICC estimates. (DOCX)
