# Supplementary material for: Evaluation of a mHealth Data Quality Intervention to Improve Documentation of Pregnancy Outcomes by Health Surveillance Assistants in Malawi: A Cluster Randomized Trial
Source: PLoS One. 2016 Jan 5;11(1):e0145238. doi: 10.1371/journal.pone.0145238 (PMC4701446; doi:10.1371/journal.pone.0145238)
Supplement: S1 File — (DOCX) [file pone.0145238.s001.docx]

**S1. Selection of SMS sent to RMM HSAs during phases one and two**

In phase one, HSAs in the control group received one of three basic motivational SMS twice a week during work hours (Table S1.1). We randomized the sequence of the three SMS every 1.5 weeks to minimize alert fatigue, the avoidance of notifications such as SMS due to frequency [34].

**Table S1.1. Control group SMS: motivational SMS in phases one and two.**

| **Phase one SMS** | **Phase two SMS** | |
| --- | --- | --- |
|  | **Updated phase one SMS** | **New SMS** |
| Good morning! The NSO^a^ thanks you for your efforts in RMM. | NSO^a^: Good morning! The NSO thanks you for your efforts in RMM. Zikomo!^b^ | NSO^a^: HSAs^c^ work very hard for RMM and the NSO recognizes your efforts. We appreciate your work! Zikomo!^b^ |
| Good Morning! Remember to complete your RMM work. | NSO^a^: Good Morning! Remember to complete your RMM work. Zikomo!^b^ | NSO^a^: HSAs^c^ have many responsibilities and each is important. The NSO recognizes your efforts in RMM! Zikomo^b^ |
| Thank you for your contribution to RMM. Have a nice day! | NSO^a^: Thank you for your contribution to RMM. Have a nice day! | NSO^a^: HSAs^c^ are dedicated to improving community health. The NSO appreciates your work. Zikomo!^b^ |

*^a^NSO - National Statistical Office*

*^b^Zikomo - Thank you in local language, Chichewa*

*^c^HSAs - Health Surveillance Assistants*

In phase one, HSAs in the treatment group received one of 12 data quality and motivational SMS three times a week during work hours. A convenience sample of six SMS is included in Table S1.2. The sequence of these messages was randomized every four weeks to avoid alert fatigue, yet reinforce important data quality guidelines. The first and third SMS each week for the treatment group coincided with the first and second SMS each week for the control group. No SMS was sent to the control group when the treatment group received the second weekly SMS.

**Table S1.2. Treatment group SMS: convenience sample of data quality and motivational SMS from phase one and improvements made for phase two.**

|  | **Phase one SMS** | **Phase one SMS** |
| --- | --- | --- |
| **Data quality** | A woman is no longer pregnant but has no baby. Have you asked about abortion, miscarriage, NND^a^, and stillbirth? Document these events in VHR^b^ & extraction forms^c^! | **NSO**^g^:A woman is no longer pregnant but has no baby. Have you asked about abortion, miscarriage, NND^a^, and stillbirth? Write these events in VHR^b^ & extraction forms^c^ |
|  | Ask the VHC^d^ about pregnancies, births, and deaths. They are a resource and support your RMM work. | **NSO**^g^: Visit your VHC^d^ often to ask about pregnancies, births, stillbirths, and deaths. They are very aware of community events and can help support your RMM work.. |
|  | A baby born alive but dies in one month is a NND^a^. Document in birth & death tables. A baby born dead is a stillbirth. Document in pregnancy table with comment. | **NSO**^g^: A baby born alive but dies in one month is a NND^a^. Document in birth & death tables. A baby born dead is a stillbirth. Write in pregnancy table with comment |
|  | You identified a death but didn’t have your VHR^b^? Remember to document the death in the VHR^b^ and extraction forms so that it counts. | **NSO**^g^: You identified a death but didn’t have your VHR^b^? Remember to write the death in the VHR^b^ and extraction forms. Stillbirths, NNDs^a^- all deaths are important! |
| **Motivational** | Are you the HSA^e^ of the quarter^f^? Find out at the January review meeting. Winners receive extra airtime. | **NSO**^g^: Are you the HSA^e^ of the quarter^f^? The NSO^g^ wants to recognize HSAs who excel in RMM. Find out at the January review meeting. Winners receive extra airtime. |
|  | Extraction forms for September 2012: Salima 77 out of 78. Balaka 69 out of 77. Good job Salima! HSAs^e^, remember to complete and submit your forms on time. | **NSO**^g^: Extraction forms for September 2012: Salima 77 out of 78. Balaka 69 out of 77. Good job Salima! HSAs^e^, remember to complete and submit your forms on time. |

*^a^NND - Neonatal death*

*^b^VHR - Village Health Register*

*^c^Extraction forms are the monthly reporting forms for RMM reporting pregnancies, births, and deaths*

*^d^VHC - Village Health Committee*

*^e^HSAs - Health Surveillance Assistants*

*^f^HSA of the quarter- a RMM incentive of additional airtime provided at quarterly data review meetings to select HSAs meeting high data quality standards*

*^g^NSO - National Statistical Office*

Since the start of the mHealth intervention, HSAs in both groups asked for more variation in SMS content, though some HSAs in the treatment group recommended repeating SMS to emphasize important data quality issues. In response to this feedback we planned and implemented phase two. For the control group we modified the three phase one SMS and added three new SMS for a total of six SMS (Table S1.1). For the treatment group we modified the 12 phase one SMS and added 12 new SMS for a total of 24 SMS for phase two (Tables S1.2 and S1.3).

**Table S1.3. Treatment group SMS: sample of new data quality SMS for phase two**

| **New phase two data quality SMS** | |
| --- | --- |
| **NSO**^a^: To address community needs you need to know the health of your community- pregnancies, births, stillbirths, & deaths. Document in VHR^b^ and extraction forms! | **NSO**^a^: Catchment areas grow & shrink with births, migration and death. Document pregnancies, births, deaths in VHR^b^ and extraction form to keep track! |
| **NSO**^a^: Dedicated HSAs^c^ know their communities. Document pregnancies, births, stillbirths, and deaths in VHR^b^ and extraction forms. Data helps with work planning! | **NSO**^a^:A family notified you of a maternal death.Did you ask about the baby? Is the baby alive? NND^d^? Stillbirth?Probe respectfully to identify and document for RMM |
| **NSO**^a^:A Family notified you of a newborn death. Probe respectfully to confirm stillbirth or NND^d^. Baby born alive but dies is a NND^d^. Baby born dead is a stillbirth | **NSO**^a^: Your VHCs^e^ may identify pregnancies, births, deaths, and stillbirths that you had not identified. Work with your VHC^e^ since they can support your work. |

*^a^NSO - National Statistical Office*

*^b^VHR - Village Health Register*

*^c^ HSAs - Health Surveillance Assistants*

*^d^NND - Neonatal death*

*^e^VHC - Village Health Committee*

We modified phase one SMS by starting the SMS with “*NSO*:” to notify the HSA of the information source immediately at the start of SMS. We ended the SMS with “*Zikomo,*” thank you in the local language, Chichewa, if the SMS was short enough to include the additional characters. Due to the 160-character limit, only a few treatment group SMS included *Zikomo*. We also increased the frequency of weekly SMS in the treatment group from three SMS a week to five SMS a week. We did not change the frequency of SMS in the treatment group.
