# Supplementary material for: Evaluation of a mHealth Data Quality Intervention to Improve Documentation of Pregnancy Outcomes by Health Surveillance Assistants in Malawi: A Cluster Randomized Trial
Source: PLoS One. 2016 Jan 5;11(1):e0145238. doi: 10.1371/journal.pone.0145238 (PMC4701446; doi:10.1371/journal.pone.0145238)
Supplement: S2 File — (DOCX) [file pone.0145238.s002.docx]

**S2. Power and ICC estimates**

Baseline data were not used in the design phase to calculate the intracluster correlation (ICC). We proposed an ICC of 0.3 for the calculation of the design effect. This was determined since HSAs work independently in the field yet are connected with HSA peers at the health facility. A level of homogeneity among HSAs in a cluster is probable, though it is not assumed to be high given the isolation in which HSAs work. We planned to check our assumption once we calculated ICC using baseline data.

In the recalculation of design effect and power, we used Stata 12 to calculate the ICC of matched pregnancies using baseline results of HSAs in all clusters [39]. We calculated an ICC of 0.015 (0.002-0.027), far smaller than the estimated ICC of 0.3 used in the design of the study (Table S2). Cluster size ranged from 1 to 19 HSAs per cluster with an average of 5.2 HSAs per cluster and 0.77 coefficient of variation of cluster sizes. We calculated a more favorable power calculation using baseline data but report the power of 0.54 as the study was designed with this power estimate [38].

**Table S2. ICC calculations: baseline and intervention periods.**

|  | **ICC** | **95% CI** |
| --- | --- | --- |
| **Baseline** | 0.015 | 0.002-0.027 |
| **Intervention** |  |  |
| *Treatment group* | 0.022 | 0.000-0.048 |
| *Control group* | 0.063 | 0.004-0.122 |

We also calculated the ICC of matched pregnancies during the intervention period, by treatment group. We calculated an ICC of 0.063 for the control group. The treatment group ICC was slightly lower during the intervention period at 0.022.
